# Adenocarcinoma of the duodenum arising from Brunner’s gland resected by partial duodenectomy: a case report

**DOI:** 10.1186/s40792-019-0732-4

**Published:** 2019-11-14

**Authors:** Tetsuya Mochizuki, Nobuaki Fujikuni, Koichi Nakadoi, Masahiro Nakahara, Kazuaki Tanabe, Shuji Yonehara, Toshio Noriyuki

**Affiliations:** 1 Department of Surgery, Onomichi General Hospital, Onomichi, Hiroshima Japan; 2 Department of Gastroenterology, Onomichi General Hospital, Onomichi, Hiroshima Japan; 3 Department of Gastroenterological and Transplant Surgery, Graduate School of Biomedical and Health Sciences, Hiroshima University, Hiroshima, Japan; 4 Department of Pathology, Onomichi General Hospital, Onomichi, Hiroshima Japan

**Keywords:** Adenocarcinoma, Brunner’s gland, Duodenal carcinoma

## Abstract

**Background:**

Duodenal carcinoma originating in Brunner’s gland is rare. Herein, we report a case of duodenal carcinoma arising from Brunner’s gland in a 63-year-old man.

**Case presentation:**

On diagnostic imaging, the lesion presented as a non-invasive carcinoma; the patient also had uncontrolled diabetes and liver cirrhosis. Hence, we decided to perform partial duodenectomy to reduce operative stress. Pathological examination revealed that the tumor consisted of tissue from Brunner’s gland. Additionally, the carcinoma cells were strongly positive for Mucin-6 protein, which is an epithelial marker of Brunner’s gland. The patient’s post-operative course was uneventful, and he has been well for 2 years after the surgery.

**Conclusions:**

This a rare case of an adenocarcinoma arising from Brunner’s gland of the duodenum that was resected by duodenectomy.

## Background

Primary adenocarcinoma of the duodenum constitutes less than 1% of all carcinomas of the gastrointestinal tract [1]. Brunner’s glands consist of submucosal mucin-secreting glands. Brunner’s glands mainly exist in the first and second portions of the duodenum [2, 3]. Since the development of modalities for investigating the upper gastrointestinal tract, the accuracy of diagnosis of carcinoma of the duodenum has increased. However, adenocarcinoma arising from Brunner’s gland has been rare.

Here, we report a rare case of a primary adenocarcinoma arising from Brunner’s gland, wherein partial duodenectomy was performed.

## Case presentation

**Fig. 1:**
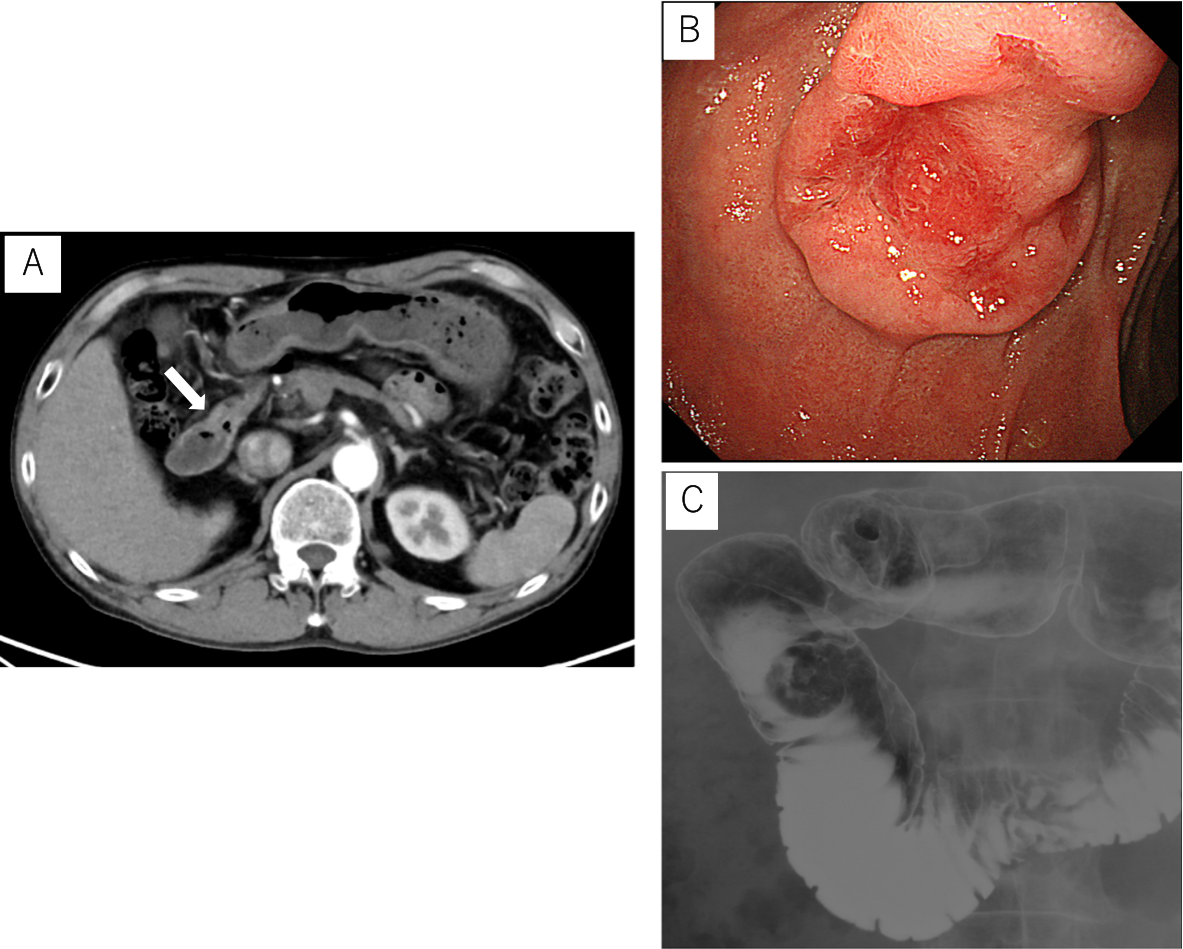
**a** An enhanced computed tomography revealed the thickened wall of the duodenum without invasion outside the wall, nor lymph node swelling (arrow). **b** Gastrointestinal endoscopy showed a type 2 tumor, measuring about 25 mm in diameter at the second portion of the duodenum. **c** An upper gastrointestinal image revealed a clearly demarcated filling defect in the second portion of the duodenum

A 63-year-old man, without any chief complaint, was referred to our hospital because of abnormal duodenal mucosa found during an upper gastrointestinal endoscopy during screening. The patient had been on medications for diabetes mellitus, diabetic neuropathy, alcoholic liver cirrhosis, and hypertension. Physical examination revealed no abnormalities. Laboratory examination results revealed liver damage B; Child-Pugh, A; and glycated hemoglobin (HbA1c), 8.4%. An enhanced computed tomography scan revealed thickening of the wall of the duodenum. There was no invasion outside the wall or lymph node swelling (Fig. 1a). Upper gastrointestinal endoscopic examination revealed a type 2 tumor in the second portion of the duodenum (Fig. 1b). Additionally, an upper gastrointestinal image revealed a clearly demarcated filling defect in the second portion of the duodenum (Fig. 1c). Finally, histological analysis of the biopsied samples revealed papillary adenocarcinoma.

**Fig. 2:**
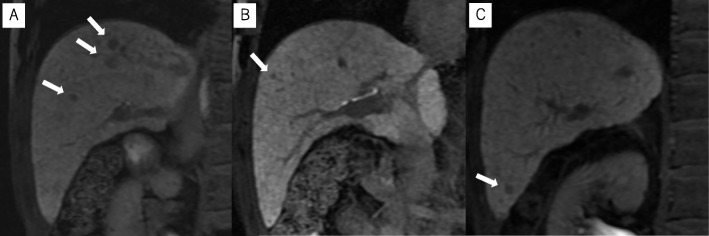
**a**–**c** Magnetic resonance imaging revealed low nodular shadows in the liver cell phase in the anterior segment and S6

Based on the imaging and pathological studies, the tumor was diagnosed as adenocarcinoma of the duodenum. A surgical duodenectomy was planned. Although pancreaticoduodenectomy was initially considered, partial duodenectomy was the final choice to reduce operative stress due to comorbidities, such as uncontrolled diabetes, liver cirrhosis, and suspected hepatocellular carcinoma (HCC) [from magnetic resonance imaging (Fig. 2)]. Additionally, the lesion was identified as a non-invasive carcinoma from the imaging findings; therefore, partial duodenectomy was preferred in this case. Radiofrequency ablation was scheduled for HCC after the surgery.

**Fig. 3:**
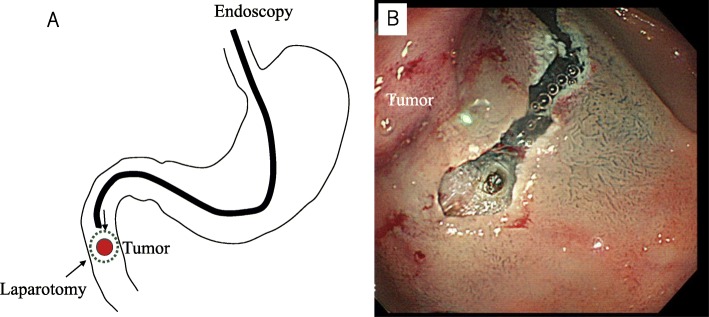
**a** Schema of the surgery. Partial duodenectomy was performed using both endoscopy and laparotomy. **b** A circumferential mucosal incision was made around the tumor using the endoscopic submucosal dissection technique

Prior to the endoscopic procedure, the jejunum was clamped using removal forceps. Tumor location was confirmed using both endoscopy and laparotomy, and the periphery of the tumor was marked by endoscopy. A circumferential mucosal incision was made around the tumor by endoscopic submucosal dissection (ESD) technique (Fig. 3b). A partial full-thickness incision was made, and seromuscular incision was performed by surgical operation along the mucosal incision line made using an endoscope technique from the full-thickness incised portion (Fig. 3a). Subsequently, the duodenal wall defect was closed with a hand-sewn suturing technique. Thereafter, the endoscope was inserted and passed over the resected location to confirm that there was no stenosis or leakage.

**Fig. 4:**
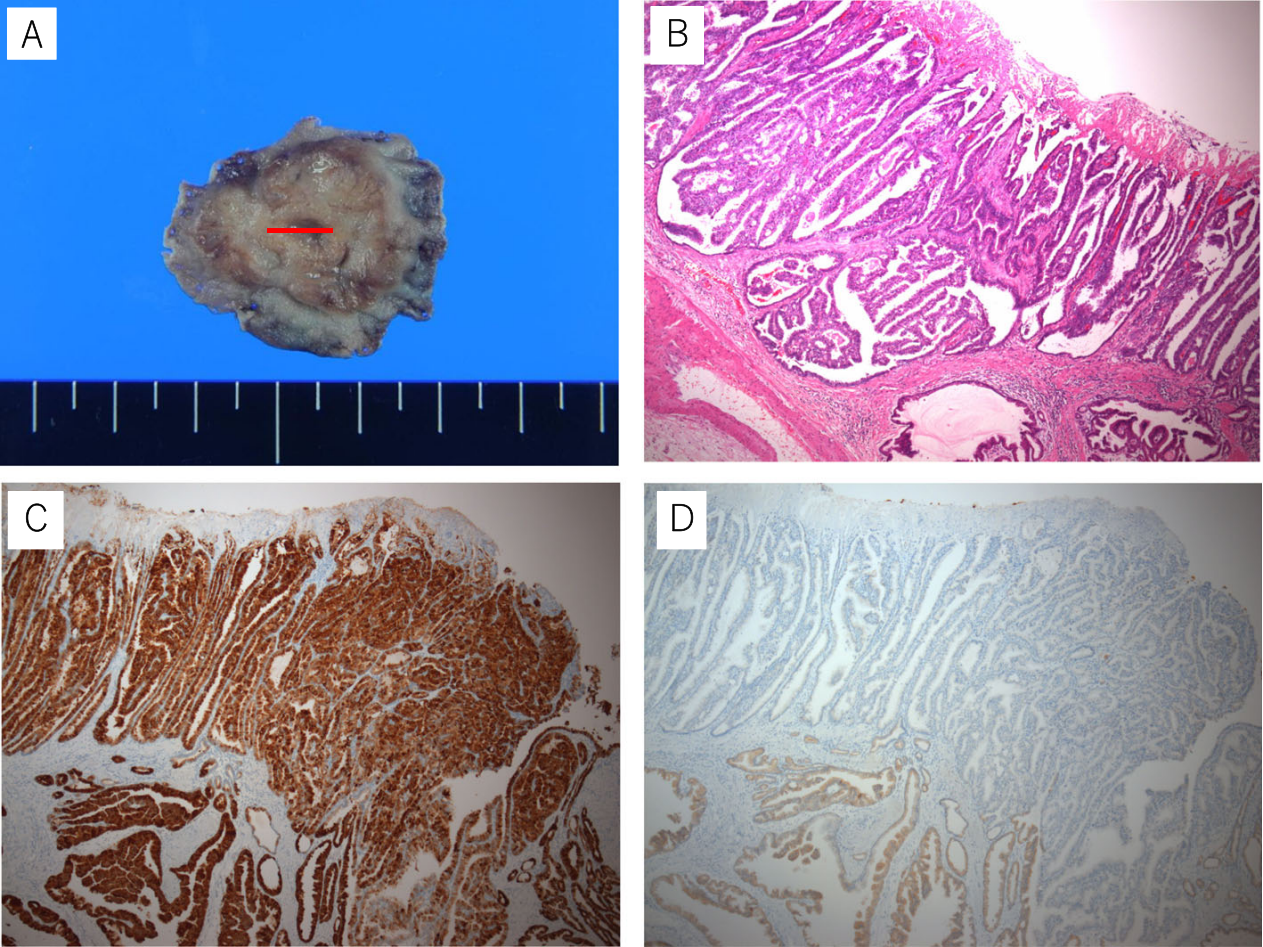
**a** The resected specimen shows submucosal tumor. A lesion with a high degree of nuclear atypia was found in the center of the tumor (red line). **b** Atypical cells with dense chromatin forming irregular glandular structure were found in the superficial layer. **c**, **d** Immunohistochemical staining revealed that the site of atypical cell was positive for MUC-6 (**c**) but negative for MUC-5 AC (**d**)

Histopathological assessment of the resected specimen revealed proliferation of the Brunner’s gland. Atypical cells with dense chromatin forming irregular glandular structure were found in the superficial layer (Fig. 4b). Immunohistochemical studies revealed that proliferated Brunner’s glands were positive for mucin-6 (MUC6) (Fig. 4c) and negative for mucin-5 AC (MUC5AC) (Fig. 4d). The tumor was 8 × 8 mm in size, with no lymphatic invasion, no venous invasion, and negative lateral and vertical margins, and the final pathological stage was IA according to the Japanese Classification of Gastric Carcinoma (T1a(M)N0 M0).

The patient did not have any specific post-operative complications; he was postoperatively discharged after 11 days. After the operation, liver tumor with alcoholic nodules was diagnosed by hepatic angiography. There was no recurrence at the 2-year follow-up.

## Discussion

Brunner’s glands are mucus-secreting acinotubular glands. They extend distally from the pylorus for a variable distance, usually stopping at the first and second portions of the duodenum and rarely stopping at the third and fourth portions [2, 3]. They consist of submucosal mucin-secreting glands exclusively located in the duodenum; therefore, proliferative Brunner’s gland lesions looks like submucosal tumors upon endoscopy [4]. Most of the lesions are hyperplasia, and adenocarcinoma arising from Brunner’s gland is rare [4–6]. The first case was reported in 1894 by Pic [7]. In 2007, Koizumi et al. summarized 21 cases of carcinoma arising from Brunner’s gland [6] and five more cases were later reported [4, 5, 8–10] (Table 1).

**Table 1:** Review of duodenal carcinoma arising from the Brunner’s glands

Age (years)	39–86 (mean 67.2)
Gender (male to female)	20:6
Location	
1st	12 (46.2%)
2nd	13 (50.0%)
3rd	1 (3.8%)
Macroscopic appearance	
SMT	8 (30.8%)
Polypoid	4 (15.4%)
Sessile	8 (30.8%)
Type 2	5 (19.2%)
Others	1 (3.8%)
Depth of invasion	
T1	16 (61.5%)
T2	1 (3.8%)
T3	3 (11.5%)
T4	2 (7.7%)
Unknown	4 (15.4%)
Operation	
Polypectomy	2 (7.7%)
EMR	4 (15.4%)
Partial duodenectomy	9 (34.6%)
Distal gastrectomy	2 (7.7%)
Pancreatoduodenectomy	6 (23.1%)
Unknown	3 (11.5%)

There is no exclusive marker for adenocarcinoma of the Brunner’s gland. Thus, the diagnosis in this case was made by histological examination. Additionally, it is difficult to diagnose by hematoxylin-eosin staining alone. Immunohistochemical examination of pyloric/Brunner’s gland-type mucin (MUC6) and gastric foveolar-type mucin (MUC5AC) is necessary to confirm the origin from Brunner’s gland. The MUC6 gene is thought to be specific for Brunner’s glands, pyloric glands, and mucus neck cells of the stomach. MUC5AC is positive in hyperplasia and negative in adenoma and adenocarcinoma [4]. In our present case, Brunner’s gland adenocarcinoma was indicated by the fact that the cancer was surrounded by Brunner’s gland hyperplasia and immunostaining analysis was positive for MUC6 and negative for MUC5AC.

The treatment strategy for adenocarcinoma of the Brunner’s gland is controversial. In case of duodenal adenocarcinoma, Kerremans et al. reported that Whipple resection should not be considered in case of an existing lymph node invasion [11]. Kerremans et al. reported the associations of survival period with presence of regional lymph node involvement. Kaklamanos et al. reported that segmental duodenal resection is associated with postoperative morbidity and long-term survival [12]. Gold et al. reported that the rate of lymph node positivity was not associated with long-term survival [13]. Jordan et al. described lymph node positivity as one of the most important prognostic factor; therefore, lymphadenectomy should be considered in such cases [1].

Adenocarcinomas of the Brunner’s gland are most frequently treated by pancreatoduodenectomy (36.0%), partial duodenectomy with gastrectomy (28.0%), or partial duodenectomy (16.0%) [8]. In 2007, Koizumi et al. reported ten cases of limited resection, comprising of six partial resections of the duodenum, two endoscopic mucosal resections, and two polypectomies [6]. Since, in our case, the patient had uncontrolled diabetes, alcoholic liver cirrhosis, and suspected HCC, we performed partial duodenectomy for duodenal tumor to reduce operative stress.

The prognosis of duodenal carcinoma is poor. Hung et al. reported the 5-year survival rate to be 7.9% [14]. The prognosis of duodenal carcinoma from the Brunner’s gland is unclear. More reports are required to build consensus on the prognosis and treatment strategy in adenocarcinoma of the Brunner’s gland.

Consequently, partial duodenectomy was successfully performed in a 63-year-old man with adenocarcinoma arising from the Brunner’s gland. The patient was discharged 11 days after the surgery. There were no specific post-operative complications or recurrence at the 2-year follow-up.

## Conclusions

This is a rare case of an adenocarcinoma arising from the Brunner’s gland of the duodenum that was resected by partial duodenectomy.

## Data Availability

The patient data for this case report will not be shared to ensure patient confidentiality.

## Abbreviations

ESD: Endoscopic submucosal dissection
HCC: Hepatocellular carcinoma
MUC5AC: Mucin-5 AC
MUC6: Mucin-6
